# Supplementary material for: Super-resolution laser probing of integrated circuits using algorithmic methods
Source: Nat Commun. 2022 Sep 2;13:5155. doi: 10.1038/s41467-022-32724-z (PMC9440222; doi:10.1038/s41467-022-32724-z)
Supplement: Supplementary file 1 — Supplementary Information [file 41467_2022_32724_MOESM1_ESM.pdf]

Supplementary Information: Super-resolution laser probing of integrated circuits using algorithmic methods

V.K. Ravikumar, J.M. Chin, W. Lua, N. Linarto, G. Ranganathan, J. Trisno, K.L. Pey*, J.K.W. Yang*

Computation of the MC map requires a transformation of the physical layout. In supplementary Figure 1, the physical layout of the cell discussed is shown. The bounding box of the cell, shown in red contains all the 24 transistors that make up the combinational logic circuitry. Each transistor is identified by the intersection of the gate (green, vertical) with a pair of fins (yellow, horizontal). There are four rows of transistors along the X axis. The mid rows contain PMOS transistors, and the extremes contain the NMOS transistors. The global signals to the cell are the three inputs (A1-A3) and its output (OUT). The rest of the signals are internal to the cell, and are wired up to perform a specific algorithmic operation. During test execution, the voltages taken by each internal net will be dependent on the combination of the input nets as well as the SPICE model of the cell, which can be extracted through available test and design information. Once the sequence of electrical fluctuations within the cell are computed, the modulation capacity map can be constructed, which is discussed in Fig. 2(a) of the main text.

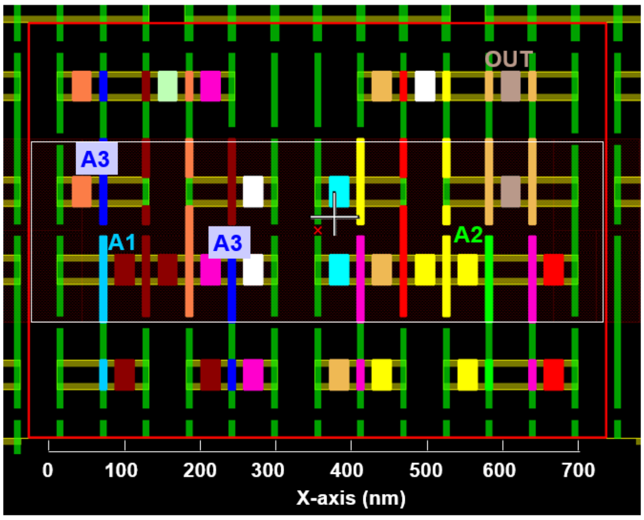

Supplementary Figure 1: physical layout of the cell of interest *(red bounding box). The vertical green polygons are the gates, and four pairs of yellow horizontal lines are the fins. The white bounding box indicates the PMOS transistors, and the fins outside this box are the NMOS transistors. The three inputs (gates) to the cell, and the output (fins) are marked*

A1, A2, A3 and OUT respectively. All other colourful polygons indicate internal signal nets to the cell. The areas of overlap between the gates and the fins indicate the functional regions of the transistors. The modulation capacity map described in Fig.2 of the main text is extracted from the physical layout and the electrical stimuli to the cell.

Supplementary information for Fig. 2 of the main text is discussed here. The PCC percentages of r1 and r2 for multiple simulated positions are shown in supplementary Table 1(a). The peak PCCs for r1 and r2 are at positions 471 (98%) and 497 (96%) respectively. The PCCs drop between 1% to 4% within 10 nm. For example, at the midpoint (position 484) or 13 nm away, the PCCs for both waveforms have dropped between 5 – 6 %, clearly distinguishing the positions. While all PCC curves in Fig. 2 (e) exhibit global maximum, the shape of their profile can be broad or narrow depending on the contrasting electrical fluctuations under the optic probe at each spot. Therefore, the probe placement resolution 'd' depends on the thresholding of the PCC curve as described in supplementary table 1(b). In the case of the curve pcc4, a threshold of 10% (10% below the peak PCC) would correlate with waveforms collected from a 91 nm wide region. Whereas, when we apply a threshold of 0.1%, we can narrow down the correlated positions to within a 9 nm wide region. Not all waveforms (for example pcc1) collected can be accurately placed, due to the limited contrast in the modulation capacity in the region that the waveforms were collected from. We also note that applying such a threshold to the gaussian model of the optic probe results in 6 nm single axis resolution at a threshold of 0.1%, which would be the practical limit of this technique for this wavelength.

Supplementary Table 1. 10 nm probe placement resolution

a. Cross correlation scores for 2 raw waveforms

Simulation position (nm)	PCC with r1 (%)	PCC with r2 (%)
**100**	42	16
**300**	46	2
**450**	92	56
**461**	96	67
**471**	**98**	78
**484**	94	91
**497**	80	**96**
**507**	69	95
**517**	53	89
**550**	6	60
**600**	-55	0

b. Resolution at tip of PCC curves

Waveform	d (10%) (nm)	d (1%) (nm)	d (0.1%) (nm)
**pcc1**	**114**	**34**	**34**
**pcc2**	**166**	**57**	**16**
**pcc3**	**75**	**27**	**10**
**pcc4**	**91**	**30**	**9**
**Optic probe**	**64**	**20**	**6**
